# Four Cases in the Philadelphia Hospital of Oral Surgery

**Published:** 1882-04

**Authors:** Jas. E. Garrettson

**Affiliations:** Surgeon in Charge


### CLINICAL REPORTS.

### FOUR CASES IN THE PHILADELPHIA HOSPITAL OF ORAL SURGERY.

CLINIC OF PROF. JAS. E. GARRETTSON, M.D., SURGEON IN CHARGE.
Reported by a Student for the INDEPENDENT PRACTITIONER.

CASE I. *Necrosis of the Lower Jaw.*—GENTLEMEN : This case, you will remember, was brought to us by Dr. J. V. Shoemaker with a view of consultation as regards the jaw of the patient, which was in a diseased condition. The doctor told us that the young man had been under his treatment for some time, and that he had found him suffering from *necrosis* of the bone. He operated, removing two sequestra, and after the removal, for the purpose of exciting granulative action, he had passed through the opening made by the operation, using as the means a probe, a tent of absorbent cotton. This is to be accepted as very judicious treatment and should have resulted successfully. Unfortunately there occurred another break in the continuity of the region, and the doctor, on examination, not being fully satisfied as to the cause of this break, brought the patient here for examination. I suggested, on looking carefully at the condition of the teeth of the locality, that two of them might be acting as irritants, these preventing cure and maintaining the discharge from the sinuses.

The teeth, gentlemen, are valuable organs, and are not to be sacrificed without reasons that arise out of something more than mere guess-work. The organs here involved are the two inferior eye-teeth. I say involved, because on examination we find the entire external alveolar boundary removed from about their roots. Nature is at work making great effort to get rid of the bodies.

Founding a conclusion on many special data possessed in the particular direction, I have no hesitation in saying that the sinuses discharging beneath the chin will continue in defiance of treatment, however carefully and scientifically conducted, until either the surgeon or the vis medicatrix naturæ pulls these teeth. The cuspidati are bad teeth to lose. Upon these bodies depend very markedly the strength of the dental arches. When, however, dead eye-teeth threaten the destruction of a jaw, the removal of them constitutes the lesser of two evils pertinently facing a practitioner having the case in charge.

We decide to extract the teeth. The patient will return in a week that the result may be seen.

The patient later reported that the sinuses had closed on the second day. The case was dismissed, cured.

CASE II. *Necrosis of the Mastoid Portion of Temporal Bone.*—This other case is also sent to us by Dr. Shoemaker. The name of the patient is Joseph R. H. His age is 20 ; his occupation is that of a ship-blacksmith. This man was accidentally struck on the side of the head by a sledge-hammer in the hands of a fellow-workman. He applied at the American Hospital for Skin Diseases, and the doctor discovered the trouble in the bone, and kindly directed the applicant here for treatment. The hearing of the patient is unaffected. Questioning him I derive the impression that the injury pertains to the superficial portion of the bone and does not extend deeply into its substance.

Now, gentlemen, it would be quite easy to say, in a case like this, that there is necrosis, but to be certain of it we will proceed to proof, and in so doing I employ this probe, which is a silver instrument you have seen me use often in cases of like nature. I pass the probe through the sinus we have here, and I can readily feel the bone. If you will be silent for one moment, you may hear distinctly the grating of the instrument on the denuded structure. There is a difference in both the sound and touch of bone in disease and in health ; otherwise expressed, of covered and uncovered bone. The instrument struck against the iron of the stove and next against the woodwork conveys the idea.

When I lectured to the class, during the preceding course, I laid stress on the manner of blood-supply to the bone of the skull ; I called attention to the fact that the plates of these bones are situated between two membranes—the dura mater on the inside, the pericranium on the outside. These membranes furnish the structure of the bone with small arteries which keep up nutrition. Now, if the outer membrane be cut or removed, denuding thus the bone, the supply of the blood to the plate of that portion is markedly interfered with, and the internal membrane will have to yield most of what is needed until nature re-establishes the equilibrium, or, if not able to do that, to remove the portion of bone which succumbs. This dead bone is what we term sequestrum. If Nature is not strong enough to remove a sequestrum, the surgeon has to assist her. How ? I will demonstrate this to you by offering an example. Take, for instance, an ulcer—a chancre, if you please. You see between the unhealthy sore and the healthy parts a distinct line—the line of contiguity. Now we wish to remove this infecting substance, and how are we to do it? We apply some caustic, as English paste or fuming nitric acid or the like, and destroy the sore thoroughly, and by so doing involve some of the surrounding tissue; we have thus changed the expression; we have killed the disease. We still see the line of contiguity, but the parts differently demark and another line is formed which we call the line of separation, and this is the distinct line which shows the extent of the slough. When the dead part comes away, we see below it a granulating surface having a florid appearance; but should we see in any portion of this surface a small pasty portion, we know our disease has not all been destroyed, and we therefore again apply means to gain such results. Just as related of the chancre, we have demarkation between healthy and dead bone. Little by little a line of separation is formed—the line of demarkation. The dead bone is separated and forms what we term the sequestrum, and which is exactly similar to what has been described as the slough. With this pathology in view, we ask ourselves: Has the sequestrum in this case separated, or is it about to separate? This we are to find out by the use of a probe. First, I will ask our patient how long it is since the injury was received. He answers, eighteen months. This is a long period, and sc I infer that either we have a sequestrum or may avoid having one. Now, I will again examine the parts, and I use for this purpose a dental excavator. This, by the way, is an instrument that should be in every surgeon's case. I find that the bone is pretty well broken up. It is breaking up with the expression of caries.

What is to be done? I will make an incision, fill up the wound with cotton, and have this packing touched daily with tincture of iodine.

In a week I will bur the carious bone off, using for the purpose the surgical engine. Now, in making the incision you must remember where the occipital and posterior auricular arteries are. I feel for the mastoid nipple as a landmark and cut obliquely backward and upward. I cut a little vessel here, and shall have my assistant, Dr. Cottrell, attempt to pick it up and tie it. You remember we take up an artery with a pair of forceps or with a tenaculum and put a ligature around it; the proper tightening of this cuts the middle and internal coats and thus accomplishes the purpose of its intention. The trouble with vessels like the one bleeding is, that they retract into the cellular tissue and thus prevent themselves being caught.

Should any secondary hemorrhage occur here we can easily control it by compression.

CASE III. *A Case where Amputation of the Fore-arm was Performed at the Clinic of March 4th*, 1882.—This case, you will remember. I performed the amputation before you. The patient belongs to my private practice. All is going remarkably well. The man has been on the streets twice since the operation. You will recall the peculiarity of the condition. The gentleman was injured in the late war, and had a paralyzed and useless hand in defiance of a most skilful attempt to serve him in the way of a resection made by Dr. Davis, of Lancaster, Pa. Nine days have passed since the date of the amputation. The ligatures have been removed from the arteries by nature, and last evening a remaining stitch was cut from the flaps. A union of this kind is very close to that which the books denominate as immediate. Were it not for the carious bone and for a tendency toward abscess in the fore-arm, which the patient says has been a trouble for years past, I should pronounce the case well enough to be dismissed. As it is, it will be the part of discretion to keep the arm under observation a few days longer. As a medicine to be taken freely, say twenty-five drops three times daily, we will prescribe the muriated tincture of iron. Iron is the antidote to breaking-down granulation and corpuscles; muriatic tincture of iron and pus are antipodes.

This patient found himself sufficiently well to leave the hospital on the fourteenth day.

CASE IV. *Median Operation for Stone in the Bladder.*—The patient now to be introduced belongs, like the last, to my private practice. You need only see the man to appreciate that we are to deal with a condition which surrounds a major operation with complications and anxieties. The health of the gentleman is in that state where bad is daily progressing into worse, and where worse threatens to reach quickly enough the superlative of the adjectivial expression. The case is an instructive one; instructive in many respects. This patient has been a great sufferer for several years. He has tried many remedies and he has had many physicians; bougies innumerable have been used, inflicting pain which, judging from my own experience with him, must have been agonizing. Diagnosis has traversed the ground from urethral stricture to Bright's disease of the kidneys. Hope and despair have alternated from zenith to nadir ; in short, the patient has been travelling a hard road.

The trouble with him is stone in the bladder. To say whether or not a man has a stone, is to sound him. Strike a stone with a piece of steel, and you get in response a click. I have had a piece of steel, in the shape of a bougie, in the man's bladder, and I have heard the click. Hearing the click, I have the diagnosis. Having the diagnosis is not, however, having the stone. How to get the stone in this particular case is a problem that has cost me much trouble in the way of drafts on both experience and judgment. Stones are removed from bladders after a variety of manners. These matters come under the two heads of Lithotomy and Lithotrity. The two common names embrace many varieties. The election of a variety, when operation is demanded, is the test of a surgeon's proficiency in this particular direction of his art.

The patient demanding this operative relief at our hands is an ex-army officer who has suffered from many accidents incident to his profession. Among these accidents one that very particularly relates with the present condition of his case refers to a perineal injury inflicted by the pommel of a Mexican saddle. The gentleman has a series of strictures arising out of the inflammation attendant on this accident. He has a urethra so diminished in calibre and so sensitive that I am confident the passage of a large bougie without narcotic precaution would present us with an exhibition of convulsions. There exists in the case a condition of bladder irritability which denies rest day and night. The man is mentally and physically depressed, and prostrated to a degree that shows him at times with the aspect of a walking corpse. The kidneys are sympathetically, not organically, affected. The microscope has, of course, been brought into requisition.

The primary conviction about our case is that the trouble associated with it lies in the presence of the stone. A deduction is that in removal of the stone we are to find a cure. I have decided to remove the stone. How shall the offence be got from the patient's bladder ? It is no open question that the "how" is to relate with the easiest means possible. Lithotrity, or crushing, offers itself in the way of solution of the question. I have here upon the table a number of the instruments known as lithotrites. All of these, as you see, are objectionable as far as the immediate case to be treated is concerned. I, or no other surgeon, could judiciously use even the smallest of them through the urethra of the patient we are considering. Again, accepting differences of opinion as to forcing, how is such a bladder to be fairly and satisfactorily washed out? Here are cannulæ, a number of them. A cannula would about as easily go through the eye of a needle as these pass the canal. Still again, let us accept the stone to be hard. Can a surgeon possess himself of an assurance as to satisfactory results where not only possibility but probability exists as to fragments being left in a bladder? In the viscus upon which we are to operate irritability has had already too long a dwelling-place. From what I have seen of the patient I am satisfied that this irritability will soon make an end of the sufferer if surgery do not make an end of it. The mode of treatment I shall adopt is that manner of lithotomy known as the median operation. Some of you offer suggestions as to the possibility of a big stone; a calculus too large to be extracted through the limited cut characterizing this operation. Here I would find neither obstruction nor difficulty; for through the opening made I would duplicate the knife and staff with lithotrity and cannula, and in the duplication I should be sure of finding an absolutely reliable means. Median lithotomy means entering the bladder on the line of the perineal raphe. It is a delicate operation, and, as I may be pardoned in suggesting, is to be attempted by him only who knows what he is about. To accomplish the performance the surgeon proceeds as will now be shown. The gentleman being at this point brought in and etherized, Prof. Garrettson passed an ordinary catheter through which he injected into the bladder three ounces of water. This instrument being withdrawn the patient was put in the lithotomy position, the body being flexed at an angle of about fifteen degrees. The next step was the passing of a staff and the intrusting of it to a reliable assistant. "I now," said the operator, "proceed to the important step of the performance. First, I introduce the index finger of the left hand into the rectum, passing the finger upward until the top is in contact both with staff and prostate gland. If here are observed the line of the urethra, where it passes into the perineum above, and the position of the finger where it disappears into the anus below, the lookers-on will not fail to recognize the triangular aspect of an anatomy amid which we work. The apex of the triangle is the point at which the staff leaves the membranous urethra and enters the prostatic portion. The base of the triangle is the raphe of the anterior perineum. To carry a knife safely from base to apex of this triangle implies that an operator combines with a reasonable amount of manipulative skill a knowledge of the anatomy involved. I enter this double-edged scalpel into the integument eight lines above the superior edge of the anus, and push it directly toward the apex of the triangle until it reaches the staff. Feeling the blade in the groove, I advance it slightly with a view of making a nick in the gland. I next withdraw the knife, cutting upward, so as to secure an external opening not quite an inch in length. Now the finger takes the place of the blade with a view of boring a way through the prostate. Note carefully a suggestion here offering. To be certain that the finger is working its way toward the bladder and not into the cellular interspace separating bladder and rectum, be sure that the digit rests upon the convex face of the staff. If this point be overlooked attribute any success met with to luck. At this moment I have my finger in the right place, but the gland is so hard that I cannot force it. Here I bring to my assistance a blunt bistoury. The probe of the instrument I pass through the natural opening of the gland until resistance ceases as it enters the bladder. Now lateralizing the blade I cut a nick in the prostate. Withdrawing the knife and reintroducing the finger, I now find myself able to enlarge the part, which I do until I feel that the viscus is entered and the stone touched. Withdrawing now the staff I introduce forceps. Now I have the stone. The handle of the instrument tells me that the foreign body is larger than I had expected to find it. I make easy traction. The stone is coming. Here it is in my hand. The operation is over."

The stone was carried to Dr. L. Wolff, chemist, who furnishes the following analysis :

The stone weighs 10 grams and 66 centigrammes, 10.66, about ℨ ij. ℈ ij., and is composed of the following ingredients, which are enumerated here in the order of prominence :

Calcium oxalate—principally.
Protine substances.
Calcium carbonate.
Ammonio-magnesium phosphate.
Ammonium urate.

The surface of the stone is rough and sand-like ; its size is that of half a walnut flattened. It is formed of concentric layers.

The patient was seen by the writer five days later apparently on the way to rapid recovery, not an unfavorable sign having shown itself. Command of the vesical sphincter was complete after fifty hours, the urine being retained or passed at convenience. One day later the urine recovered its natural passage. The union may be termed a healing by first intention.

In two weeks the patient was again seen. He walks about the ward and is expected to return to his home in a few days.

